# Tissue-Specific Differences in DNA Modifications (5-Hydroxymethylcytosine, 5-Formylcytosine, 5-Carboxylcytosine and 5-Hydroxymethyluracil) and Their Interrelationships

**DOI:** 10.1371/journal.pone.0144859

**Published:** 2015-12-14

**Authors:** Daniel Gackowski, Ewelina Zarakowska, Marta Starczak, Martyna Modrzejewska, Ryszard Olinski

**Affiliations:** Department of Clinical Biochemistry, Nicolaus Copernicus University, Collegium Medicum in Bydgoszcz, Bydgoszcz, Poland; The Babraham Institute, UNITED KINGDOM

## Abstract

**Background:**

Replication-independent active/enzymatic demethylation may be an important process in the functioning of somatic cells. The most plausible mechanisms of active 5-methylcytosine demethylation, leading to activation of previously silenced genes, involve ten-eleven translocation (TET) proteins that participate in oxidation of 5-methylcytosine to 5-hydroxymethylcytosine which can be further oxidized to 5-formylcytosine and 5-carboxylcytosine. Recently, 5-hydroxymethylcytosine was demonstrated to be a relatively stable modification, and the previously observed substantial differences in the level of this modification in various murine tissues were shown to depend mostly on cell proliferation rate. Some experimental evidence supports the hypothesis that 5-hydroxymethyluracil may be also generated by TET enzymes and has epigenetic functions.

**Results:**

Using an isotope-dilution automated online two-dimensional ultra-performance liquid chromatography with tandem mass spectrometry, we have analyzed, for the first time, all the products of active DNA demethylation pathway: 5-methyl-2′-deoxycytidine, 5-hydroxymethyl-2′-deoxycytidine, 5-formyl-2′-deoxycytidine and 5-carboxyl-2′-deoxycytidine, as well as 5-hydroxymethyl-2′-deoxyuridine, in DNA isolated from various rat and porcine tissues. A strong significant inverse linear correlation was found between the proliferation rate of cells and the global level of 5-hydroxymethyl-2′-deoxycytidine in both porcine (R^2^ = 0.88) and rat tissues (R^2^ = 0.83); no such relationship was observed for 5-formyl-2′-deoxycytidine and 5-carboxyl-2′-deoxycytidine. Moreover, a substrate-product correlation was demonstrated for the two consecutive steps of iterative oxidation pathway: between 5-hydroxymethyl-2′-deoxycytidine and its product 5-formyl-2′-deoxycytidine, as well as between 5-formyl-2′-deoxycytidine and 5-carboxyl-2′-deoxycytidine (R^2^ = 0.60 and R^2^ = 0.71, respectively).

**Conclusions:**

Good correlations within the substrate-product sets of iterative oxidation pathway may suggest that a part of 5-formyl-2′-deoxycytidine and/or 5-carboxyl-2′-deoxycytidine can be directly linked to a small portion of 5-hydroxymethyl-2′-deoxycytidine which defines the active demethylation process.

## Background

Cytosine methylation, usually at CpG dinucleotides, is one of the most important epigenetic modifications, which has a profound impact on gene repression, cellular identity and organismal fate [1]. The reversion of DNA methylation process (demethylation) is equally important to activate previously silenced genes, and some evidence suggests that replication-independent active/enzymatic demethylation/iterative oxidation may be an important process in the functioning of somatic cells [2,3].

The most plausible mechanisms of active 5-methylcytosine (5-mC) demethylation/iterative oxidation–involve ten eleven translocation (TET) proteins that participate in oxidation of 5-mC to 5-hydroxymethylcytosine (5-hmC) which can be further oxidized to 5-formylcytosine (5-fC) and 5-carboxylcytosine (5-caC). Then BER pathway is activated by involvement of TDG glycosylase, to replace the above mentioned base modifications (5-fC, 5-caC) with cytosine and to demethylate DNA (review in [2]). Some experimental evidence supports the hypothesis that 5-hydroxymethyluracil (5-hmU) may also be generated by TET enzymes and has epigenetic functions [4].

Recently, 5-hmC was demonstrated to be a relatively stable modification, and the previously observed substantial differences in the level of this modification in various murine tissues were shown to depend mostly on cell proliferation rate [5]. Bachman et al. found a linear correlation between the proliferation rate of cells and the global level of 5-hmC in several murine tissues. Using similar tissues (brain, kidney, lung, heart, liver, muscle, spleen, gut and thymus) as in Bachman et al.’s experiments, we analyzed for the first time whether a similar relationship exists for other products of active DNA demethylation/iterative oxidation, namely 5-fC, 5-caC and 5-hmU. We used the tissues from different mammalian species, pigs and rats, whose biological age resembled that of mice being a subject of Bachman et al.’s experiments. We also looked for a potential relationship between 5-mC and its all oxidation products. To measure all the modifications mentioned above, we have developed a rapid, specific and sensitive isotope-dilution automated online two-dimensional ultra-performance liquid chromatography with tandem mass spectrometry (2D-UPLC-MS/MS).

## Materials and Methods

### Ethics statement

The study was approved by the Local Ethics Committee for Animal Experiments, and was conducted according to the rules for animal experiments, compatible with EU guidelines (2010/63/EU).

### Standards preparation

Non-labelled, genuine 5-methyl-2’-deoxycytidine (5-mdC), 5-hydroxymethyl-2′-deoxycytidine (5-hmdC), 5-formyl-2′-deoxycytidine (5-fdC), 5-carboxyl-2′-deoxycytidine (5-cadC) and 5-hydroxymethyl-2’-deoxyuridine (5-hmdU) were purchased from Berry & Associates; dA, dC, dT and dG were purchased from Sigma. Stable-isotope-labelled internal standards of [^15^N-U,^13^C-U]-2′-deoxythymidine were purchased from Cambridge Isotope Laboratories, [D_3_]-5-hydroxymethyl-2'-deoxycytidine from Toronto Research Chemicals (Toronto, Canada). [^15^N_2_,^13^C_10_]-5-mdC was synthesized using the optimized method described by Divakar and Reese [6], with [^15^N-U,^13^C-U]-2′-deoxythymidine as a substrate. Briefly, in the first step hydroxyl groups of the substrate were acetylated with acetic anhydride (Sigma-Aldrich) (3h, 20°C). After drying in vacuum concentrator, the acetylated substrate was treated with tri(1H-1,2,4-triazol-1-yl)phosphine oxide prepared freshly from 1,2,4-triazole, POCl_3_ and trimethylamine (all from Sigma-Aldrich) (45 min, 20°C), dried in vacuum concentrator, dissolved in dichloromethane and extracted with saturated NaHCO_3_. In the last step, the compound was treated with ammonia methanolic solution which replaced triazole substituent and deprotected hydroxyl groups. Resultant [^15^N_2_,^13^C_10_]-5-mdC was chromatographically purified (Luna C18 250 mm×10 mm, 5 μm, Phenomenex in 0.5% acetate—acetonitrile gradient, 0.5–15%) and characterized with LC/MS/MS. [^15^N_2_,^13^C_10_]-5-mdC, as well as [^15^N-U,^13^C-U]-2′-deoxythymidine, were further oxidized with Na_2_S_2_O_8_ (Sigma-Aldrich) (25 mg/mL in 0.1 M phosphate buffer pH 7.0) to obtain [^15^N_2_,^13^C_10_]-5-fdC and [^15^N_2_,^13^C_10_]-5-cadC (12-min reaction at 60°C) and [^15^N_2_, ^13^C_10_]-5-hmdU (20-min reaction at 60°C), respectively, using optimized method proposed by Rahman et al. [7]. All synthesized internal standards were purified with preparative HPLC (Luna C18 250 mm×10 mm, 5 μm, Phenomenex in 0.5% acetate—acetonitrile gradient, 0.5–15%) and subjected to UPLC-MS/MS and UV analyses.

Stock solutions of all standards (4 mM for dT, dA, dG and dC, 0.1 mM for 5-mdC and 0.05 mM for the others) were prepared in MilliQ-grade water. Concentrations of all standards were measured with a UV spectrophotometer, and calculated by using respective molar extinction coefficients.

### Animal tissues

The thymus, liver, gut, heart, muscle, kidney, spleen, lymph nodes and brain were obtained from three 1-year-old pigs from a local slaughterhouse. The thymus, liver, gut, heart, kidney, lung and brain were obtained from three 6-month-old male Wistar rats, kindly provided by Prof. Tomasz Drewa and co-workers from the Chair of Regenerative Medicine, Department of Tissue Engineering, Collegium Medicum in Bydgoszcz, Nicolaus Copernicus University. The pigs were sacrificed by exsanguination after electrical stunning, and the rats by lethal dose of diethyl ether. All tissues were collected immediately after the animal's death, frozen in liquid nitrogen and stored at -75°C. All animal handling protocols were approved by the Local Ethics Committees for Animal Experiments.

### DNA extraction and DNA hydrolysis to deoxynucleosides

DNA from animal tissues was isolated using the previously described method [8], with some modifications for DNA hydrolysis to deoxynucleosides. Isolated DNA was dissolved in 100 mM ammonium acetate (Sigma-Aldrich) containing 0.1 mM ZnCl_2_ (pH 4.3). The dissolved DNA samples (50 μl) were mixed with 1U of nuclease P1 (Sigma-Aldrich) and tetrahydrouridine (Calbiochem) (as cytidine deaminase inhibitor, 10 μg per sample) and incubated at 37°C for 1 h. Subsequently, 12 μl 5% (v/v) NH_4_OH (JT Baker) and 1.3U of alkaline phosphatase (Sigma-Aldrich) were added to each sample following 1-h incubation at 37°C. Finally, all DNA hydrolysates were acidified with CH_3_COOH (Sigma-Aldrich) (to final v/v concentration of 2%) and ultrafiltered prior to injection.

### 2D-UPLC–MS/MS analysis

DNA hydrolysates were spiked with a mixture of internal standards in 4:1 volumetric ratio (10 fmols for 5-cadC, 50 fmols for 5-hmdU, 500 fmols for 5-fdC and 2.5 pmols for 5-hmdC). Chromatographic separation was performed with the Waters Acquity 2D-UPLC system with a photo-diode array detector (PDA) for the first dimension chromatography, and Xevo TQ-S tandem quadrupole mass spectrometer for the second dimension chromatography. At-column dilution technique was used between the first and second dimension to improve the retention at a trap/transfer column. The following columns were used: Phenomenex Kinetex C-18 (150 mm×2.1 mm, 1.7 μm) at the first dimension, Waters X-select C18 CSH (150 mm×2.1 mm, 1.7 μm) at the second dimension, and Waters X-select C18 CSH (30 mm×2.1 mm, 3.5 μm) as a trap/transfer column. The chromatographic system operated in a heart-cutting mode, so selected parts of effluent from the first dimension were directed to the trap/transfer column via a 6-port valve switch, which served as an “injector” for the second dimension chromatography system. Flow rate and injection volume for the first dimension were 0.25 mL/min and 0.5–2 μL, respectively. The injection volume was adjusted to the quality/concentration of DNA hydrolysates, to assure that it does not exceed 2 μg of DNA per injection to prevent column overload. Typical amount of DNA injected on the column ranged between 1 μg and 2 μg. The separation was performed with a 10-min gradient elution, using a mobile phase 0.1% acetate (Sigma-Aldrich) (A) and acetonitrile (Fluka) (B) (1–5% B for 5 minutes, column washed with 30% acetonitrile and re-equilibrated with 99% A for 3.6 minutes). Flow rate at the second dimension was 0.35 mL/min. The separation was performed with a 10-min gradient elution, using a mobile phase 0.01% acetate (A) and methanol (Fluka) (B) (4–50% B for 4 minutes, isocratic flow of 50% B for 1.5 minutes, and re-equilibration with 96% A up to next injection).

The amounts of unmodified nucleosides, as well as the amount of 5-mdC after the first dimension were determined by UV detection at 280 nm for dA and dT and 300 nm for dC, dG and 5-mdC. In the case of the other compounds, mass spectrometric detection was performed using the Waters Xevo TQ-S tandem quadrupole mass spectrometer, equipped with an electrospray ionization source. The parameters of the common detector were as follows: source temperature 150°C, nitrogen desolvatation gas flow 1000 L/h, nitrogen cone gas flow 200 L/h, desolvatation temperature 500°C, and nebulizer gas pressure 7 bar. Collision-induced dissociation was obtained using argon 6.0 at 3 x 10^−6^ bar pressure as the collision gas. Transition patterns for all the analyzed compounds, as well as specific detector settings determined using the MassLynx 4.1 Intelli-Start feature, are summarized in Table 1, whereas the compound-specific validation parameters are presented in Table 2. All samples were analyzed in three to five technical replicates, and technical mean values were used for further calculations. Representative chromatograms of real samples are depicted in S1–S5 Figs.

**Table 1:** Transition patterns and specific detector settings for all analyzed compounds.

compound name		ionization mode	nominal molecular mass (Da)	pseudo-molecular ion formulation	nominal parent ion (Da)	nominal daughter ion (Da)	capillary (kV)	cone (V)	collision (eV)
5-hydroxymethyl-2'-deoxycytidine	quantifier	ESI+	257	[M+H]^+^	258	124	1.2	15	10
	qualifier	ESI+	257	[M+H]^+^	258	142	1.2	15	10
[D_3_]-5-hydroxymethyl-2'-deoxycytidine	quantifier	ESI+	260	[(M+3)+H]^+^	261	127	1.2	15	10
	qualifier	ESI+	260	[(M+3)+H]^+^	261	145	1.2	15	10
5-carboxyl-2'-deoxycytidine	quantifier	ESI-	271	[M-H]^-^	270	110	3.5	20	20
	qualifier	ESI-	271	[M-H]^-^	270	93	3.5	20	20
[^13^C_10_, ^15^N_2_]-5-carboxyl-2'-deoxycytidine	quantifier	ESI-	283	[(M+12)-H]^-^	282	116	3.5	20	20
	qualifier	ESI-	283	[(M+12)-H]^-^	282	99	3.5	20	20
5-hydroxymethyl-2'-deoxyuridine	quantifier	ESI-	258	[M-H]^-^	257	124	3.5	20	15
	qualifier	ESI-	258	[M-H]^-^	257	214	3.5	20	10
[^13^C_10_, ^15^N_2_]-5-hydroxymethyl-2'-deoxyuridine	quantifier	ESI-	270	[(M+12)-H]^-^	269	131	3.5	20	15
	qualifier	ESI-	270	[(M+12)-H]^-^	269	224	3.5	20	10
5-formyl-2'-deoxycytidine	quantifier	ESI-	255	[M-H]^-^	254	121	3.5	28	18
	qualifier	ESI-	255	[M-H]^-^	254	138	3.5	28	18
[^13^C_10_, ^15^N_2_]-5-formyl-2'-deoxycytidine	quantifier	ESI-	267	[(M+12)-H]^-^	266	128	3.5	28	18
	qualifier	ESI-	267	[(M+12)-H]^-^	266	145	3.5	28	18

**Table 2:** Compound-specific validation parameters.

	5-hmdC	5-fdC	5-cadC	5-hmdU
amount spiked (per injection)	2 pmols	50 fmols	10 fmols	50 fmols
recovery [%]	108%	93%	127%	103%
amount spiked (per injection)	10 pmols	500 fmols	50 fmols	500 fmols
recovery [%]	90%	97%	85%	105%
sample to sample RSD, n = 3	3.7%	4.7%	8.2%	10.0%
within sample RSD, n = 5	3.6%	7.5%	6.1%	6.4%
matrix factor	0.993	0.901	0.891	0.957
absolute LOD [fmols]	3	0.3	0.05	0.5
relative LOD	0.12/10^6^dN	0.01/10^6^dN	2/10^9^dN	0.02/10^6^dN
absolute LOQ [fmols]	10	0.8	0.13	1.5
relative LOQ	0.4/10^6^dN	0.03/10^6^dN	5.2/10^9^dN	0.06/10^6^dN

### Statistical analysis

**Fig 1:**
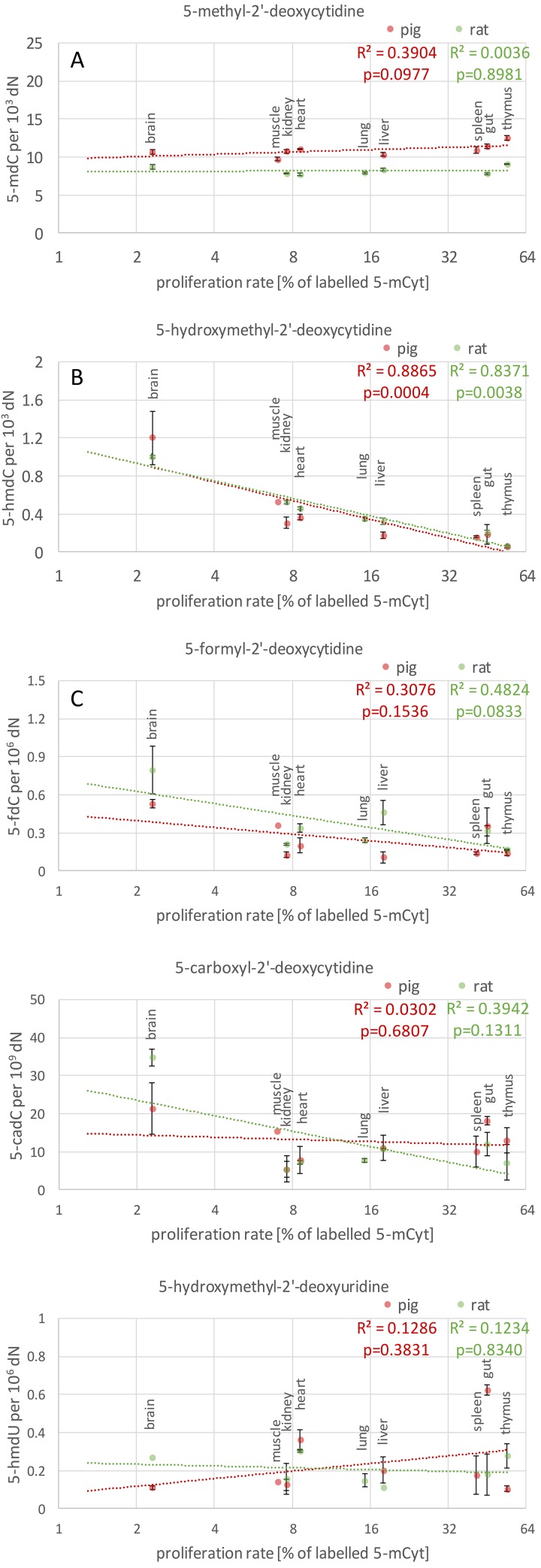
Relationship between tissue-specific proliferation rate and epigenetic DNA modifications. Relationship between tissue-specific proliferation rate, determined in a murine model and expressed as the percentage of labelled 5-methylcytosine formed in DNA during cellular division (data from [5] kindly supplied by prof. Bachman) and the content of 5-methyl-2’-deoxycytidine and active demethylation products in DNA isolated from various porcine (red) and rat (green) tissues. Coefficients of determination were calculated using a linearized regression model with logarithmic fit for proliferation rate and linear fit for all other variables. Data presented as mean values for biological replicates; error bars correspond to standard deviations.

**Fig 2:**
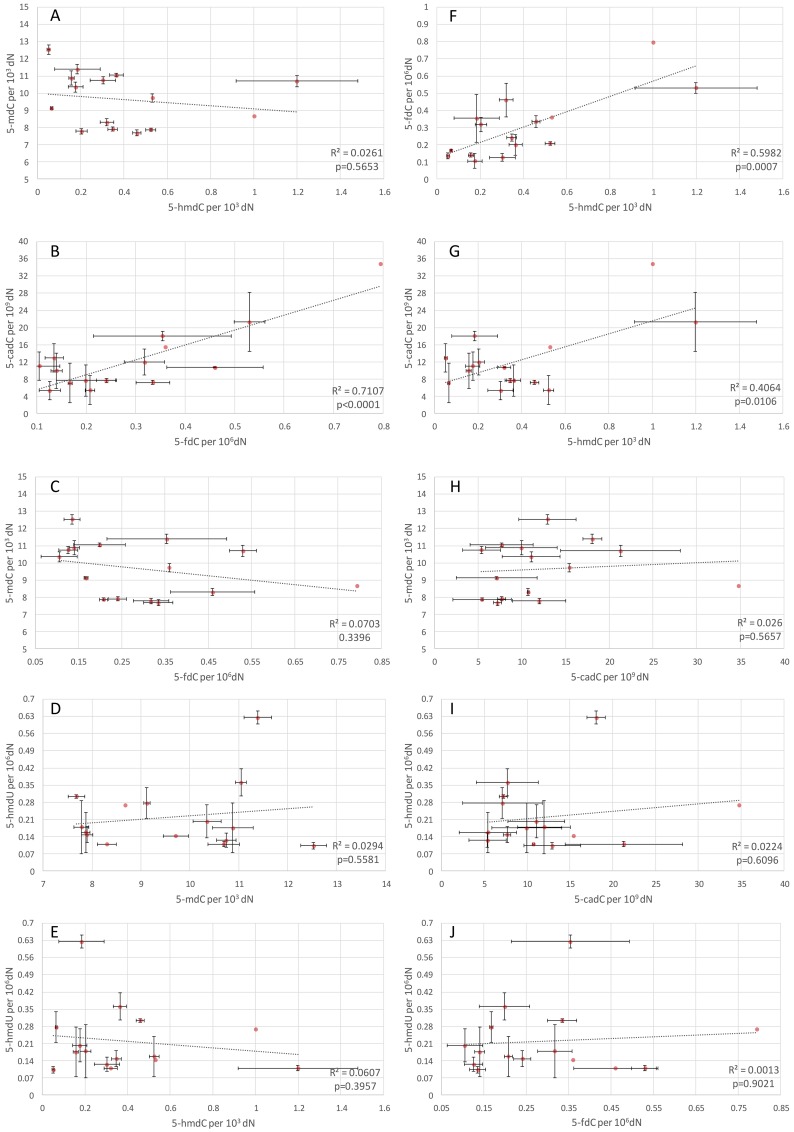
Interrelationships between DNA modifications. Interrelationships between 5-methyl-2’-deoxycytidine, 5-hydroxymethyl-2’-deoxycytidine, 5-formyl-2’-deoxycytidine, 5-carboxyl-2’-deoxycytidine and 5-hydroxymethyl-2’-deoxyuridine in DNA of various porcine and rat tissues. Coefficients of determination and p-values were calculated using Pearson’s analysis of correlation. Data is presented as mean values for biological replicates; error bars correspond to standard deviations.

Biological means of the results from animals of each species (summarized in S1 and S2 Tables) were used to determine Pearson’s correlation coefficients, describing interrelationships between the analyzed epigenetic modifications. Relationship with a proliferation rate for specific organs, determined in a murine model and described elsewhere [5], was analyzed on the raw dataset kindly provided by Dr. M. Bachman, using Statistica 12.5 linearized regression model with logarithmic fit for proliferation rate and linear fit for all other variables. All the relationships are depicted on Figs 1 and 2, along with their regression/correlation curves, coefficients of determination (R^2^) and p-values. In all cases, p<0.05 was considered statistically significant.

## Results and Discussion

Although many previous studies centered around the determination of 5-hmC level, only few authors analyzed concentrations of 5-fC, 5-caC and 5-hmU in various tissues [4,9–14]. Low abundance of the aforementioned modifications in mammalian genome (approximately 3–4 orders of magnitude lower than for 5-hmC) makes accurate determination of their levels somehow challenging.

As mentioned above, recent data clearly demonstrate that huge inter-tissue differences in 5-hmC level reflect mostly the cell proliferation status. However, it is unclear whether a similar rule refers also to other modifications. To provide a better insight into the problem in question, we analyzed 5-hmdC and other modifications using a 2D-UPLC-MS/MS method with isotopically labelled internal standards. The advantages of this technique include relatively short run times, as well as the possibility to obtain exceptionally high sensitivity and selectivity without compromising quality and validation criteria. Furthermore, fully automated two-dimensional separation is an extremely useful technique for the analyses of biological samples with mass spectrometry- based methods. It is that using this method, we were able to detect all the modifications in each type of analyzed tissue.

Assuming that the proliferation rate of cells from the same murine, rat and porcine tissue (with comparable biological age) is similar, we plotted the background levels of 5-hmdC in genomic DNA against the proliferation rate of the tissue (data kindly provided by Dr. Martin Bachman). A strong significant linear correlation between the cell proliferation rate (expressed on a logarithmic scale) and the global level of 5-hmdC was demonstrated for both porcine and rat tissues (Fig 1B). This relationship resembled closely the one reported previously by Bachman et al. To the best of our knowledge, we were the first to analyze 5-fdC, 5-cadC and 5-hmdU levels in the same set of tissues. No relationship similar to the one described above was found for these modifications (Fig 1C, 1D and 1E), which implies that levels of these compounds are independent from the cell proliferation rate (it should be mentioned, however, that the relationship for 5-fdC in rats was at a threshold of statistical significance, see Fig 1C). The levels of 5-fdC and 5-hmdU in most analyzed tissues were approximately three orders of magnitude lower than the levels of 5-hmdC, and the level of 5-cadC was 10-fold lower than the concentration of 5-fdC. Moreover, we found a considerable (20-fold) difference in 5-hmdC levels in porcine brain and thymus (characterized by the highest and lowest concentration of this compound, respectively). However, the differences in 5-fdC and 5-cadC levels in the same tissues were markedly less prominent: four- and two-fold, respectively (Fig 1C and 1D).

5-fC and 5-caC may inhibit DNA replication which may result in genome instability and mutagenesis [15,16]. Therefore, specific and effective enzymatic systems are needed to remove these modifications from DNA [17,18], and it is the activity of such systems which may explain the low level of these modifications. Moreover, 5-caC was postulated to be directly converted to cytosine *via* decarboxylation process [19], which further explains the lowest level of this modification. Another potential reason behind the markedly higher level of 5-hmC in comparison to other modifications is higher reaction rate of TET enzymes responsible for oxidation of 5-mC as compared to those involved in 5-hmC and 5-fC oxidation [20,21,22].

Since all the modifications are generated by the same enzymes (TETs), a question arises about a potential relationship between their formation and expression. To address this question, we analyzed interrelationships between the modifications (Fig 2). As expected, no correlation was found between 5-mdC and its demethylation product, 5-hmdC (the level of 5-mdC is relatively stable while the level of 5-hmdC varies substantially–see above) (Fig 2A). Therefore, we confirmed that demethylation is not the only function of 5-hmC, and that this modification is an epigenetic mark distinct from 5-mC (reviewed in [21]). However, a strong substrate-product correlation was found for another two consecutive steps of the demethylation pathway i.e. between 5-hmdC and its product 5-fdC (Fig 2F), as well as between 5-fdC and 5-cadC (Fig 2B), with the respective R^2^ values of 0.60 and 0.71. Since 5-hmdC present in DNA evidently exerts no adverse effects, the cells may be able to maintain its high levels in their genomes, efficiently removing other potentially mutagenic modifications. The evidence of high level of 5-hmC points also to the tissue- specific epigenetic role of this modification [5].

As mentioned above, 5-hmC is predominantly a stable DNA modification. However, most previous studies showed that also TET- and BER- dependent demethylation pathways are involved in the erasure of DNA methylation (as reviewed in [21,23]). Even considering the lack of a direct evidence, the presence of good correlations within consecutive substrate-product sets of the iterative oxidation pathway could imply that a portion of 5-hmC is “sealed” (destined) for the demethylation process.

Although TET enzymes are responsible for the formation of all analyzed modifications, it is still unclear how their activity is regulated, i.e. which factors decide that the oxidation of 5-mC stops at 5-hmC or proceeds to 5-fC and 5-caC. Perhaps, TET proteins show different affinity to 5-mC, 5-hmC and 5-fC (for review see [21]), or these can be different proteins (readers) that recognize the modifications and determine their fate; e.g. UHRF2 protein can specifically bind to 5-hmC and enhances its processing by TET enzymes [24].

No correlations were found for 5-hmdU irrespective of the analyzed setting (Fig 2D, 2E, 2I and 2J). Recently, it was demonstrated that 5-hmU can be also generated by TET enzymes from thymine during mouse embryonic cell differentiation [4]. Pfaffender et al. showed that the level of 5-hmU may undergo changes during epigenetic reprogramming of the cells, strictly following the patterns for other products of TET enzymes [4]. However, formation of the aforementioned modification was shown to be independent from 5-hmC deamination [4], which may explain the lack of correlations for 5-mC oxidation products.

To summarize, the results of this study suggest that irrespective of the species, 5-hmC level depends mostly on the cell proliferation status. However, other modifications involved in the process of active DNA demethylation do not follow the same rule. Good correlations within the substrate-product sets of active DNA demethylation pathway suggest that a part of 5-fC and 5-caC may be directly linked to a small portion of 5-hmC which defines the active demethylation process. However, it should be also remembered that 5-fC and 5-caC may act as dynamic epigenetic marks [25] and serve as transcription regulators [26]. Moreover, a recent study showed that the main part of 5-fC may remain stable [27]. Consequently, it is likely 5-caC level which better characterizes the 5-hmC portion destined for the demethylation process.

## Conclusions

5-hmdC is the only modification the level of which depends mostly on the cell proliferation status, irrespective of the species origin. There is no link between the levels of other modifications involved in active DNA demethylation and the cell proliferation rate. A good correlations within the substrate-product sets of active DNA demethylation pathway imply that a part of 5-fdC and 5-cadC may be directly linked to a small portion of 5-hmdC which defines the active demethylation process.

## Supporting Information

S1 Fig Sample UV chromatograms. Sample chromatograms of UV traces at 280 and 300 nm obtained in a 1D mode used to determine the amount of unmodified deoxynucleosides and 5-methyl-2’-deoxycytidine.
(PDF)

S2 Fig Sample MS/MS chromatograms of 5-hydroxymethyl-2’-deoxycytidine. Sample chromatograms of extracted MRM traces for samples with medium (left side) and low (right side) concentrations of 5-hydroxymethyl-2’-deoxycytidine as well as 2.5 pmols of [D_3_]-5-hydroxymethyl-2’-deoxycytidine.
(PDF)

S3 Fig Sample MS/MS chromatograms of 5-formyl-2’-deoxycytidine. Sample chromatograms of extracted MRM traces for samples with medium (left side) and low (right side) concentrations of 5-formyl-2’-deoxycytidine as well as 500 fmols of [^13^C_10_, ^15^N_2_]-5-formyl-2’-deoxycytidine.
(PDF)

S4 Fig Sample MS/MS chromatograms of 5-carboxyl-2’-deoxycytidine. Sample chromatograms of extracted MRM traces for samples with medium (left side) and low (right side) concentrations of 5-carboxyl-2’-deoxycytidine as well as 10 fmols of [^13^C_10_, ^15^N_2_]-5-carboxyl-2’deoxycytidine.
(PDF)

S5 Fig Sample MS/MS chromatograms of 5-hydroxymethyl-2’-deoxyuridine. Sample chromatograms of extracted MRM traces for samples with medium (left side) and low (right side) concentrations of 5-carboxyl-2’-deoxycytidine as well as 50 fmols of [^13^C_10_, ^15^N_2_]-5 hydroxymethyl-2’-deoxyuridine.
(PDF)

S6 Fig Response curves for mass spectrometric detection. The curves were generated as correlation of detector response (defined as ratio of areas under peaks of non-labelled and stable isotope-labelled compound and molar ratio of compounds in the sample. Data presented as means for two injections; error bars represent standard deviations. Coefficients of determinations and p-values were calculated using Pearson’s analysis of correlation.
(PDF)

S1 Table Individual results, biological means and standard deviations for porcine tissues. (PDF)

S2 Table Individual results, biological means and standard deviations for rat tissues. (PDF)
