# Supplementary figures and images for: Tissue-Specific Differences in DNA Modifications (5-Hydroxymethylcytosine, 5-Formylcytosine, 5-Carboxylcytosine and 5-Hydroxymethyluracil) and Their Interrelationships
Source: PLoS One. 2015 Dec 14;10(12):e0144859. doi: 10.1371/journal.pone.0144859 (PMC4682766; doi:10.1371/journal.pone.0144859)

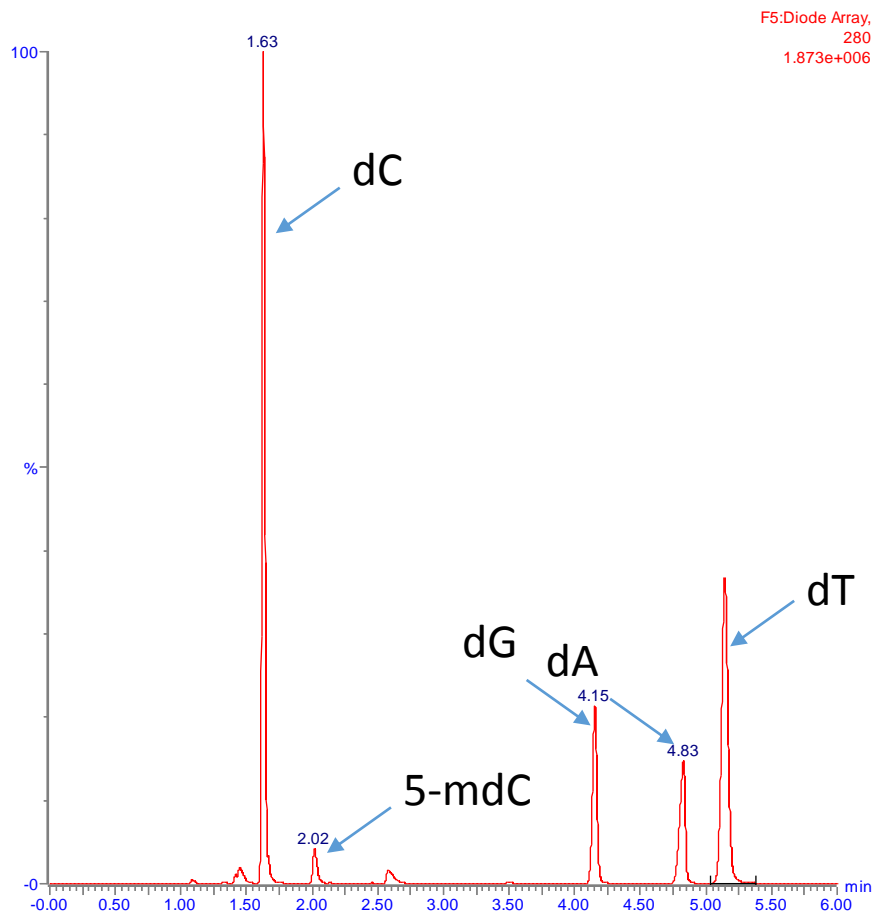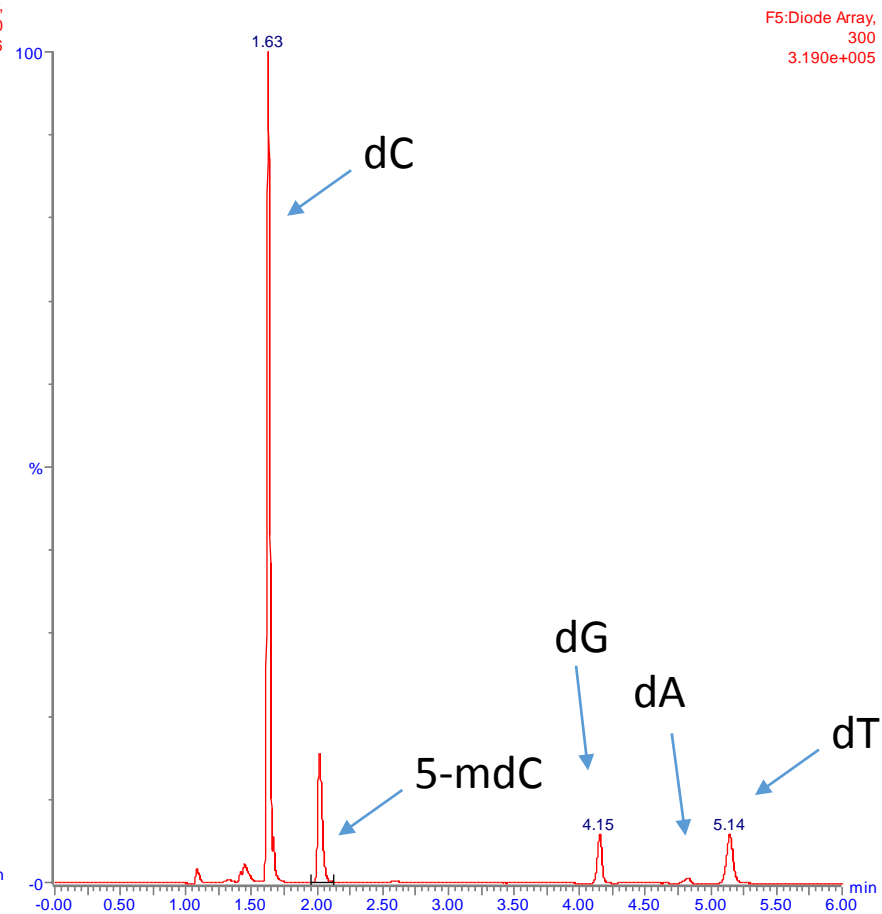

Supplement: S1 Fig — Sample chromatograms of UV traces at 280 and 300 nm obtained in a 1D mode used to determine the amount of unmodified deoxynucleosides and 5-methyl-2’-deoxycytidine. (PDF) [file pone.0144859.s001.pdf]

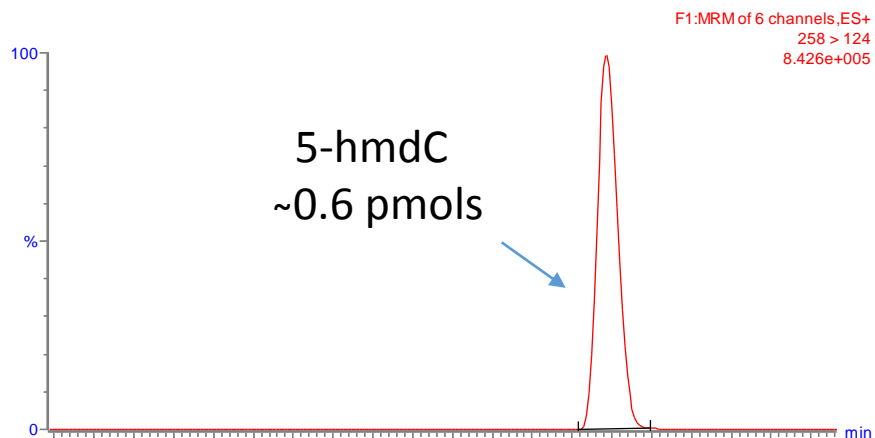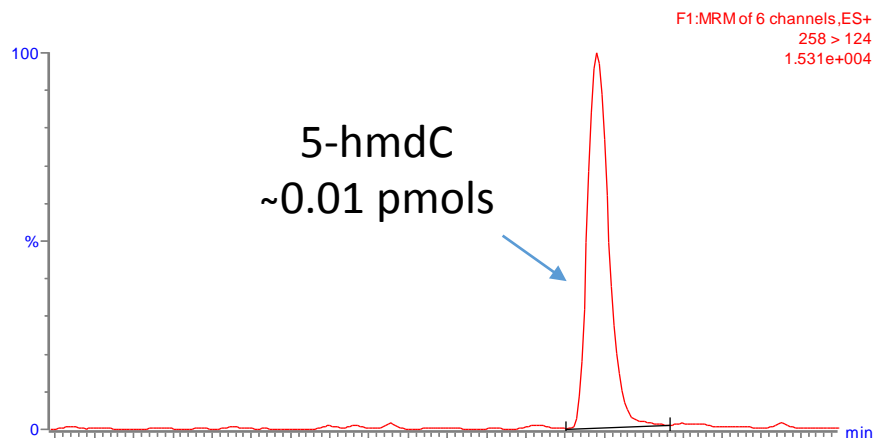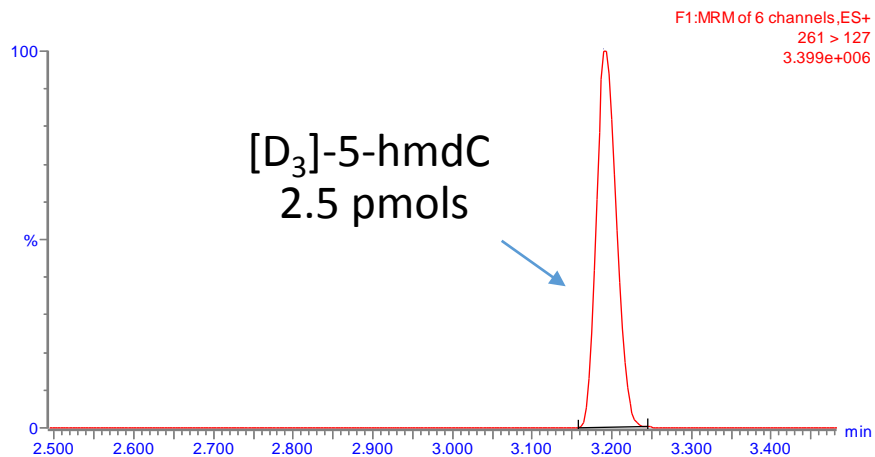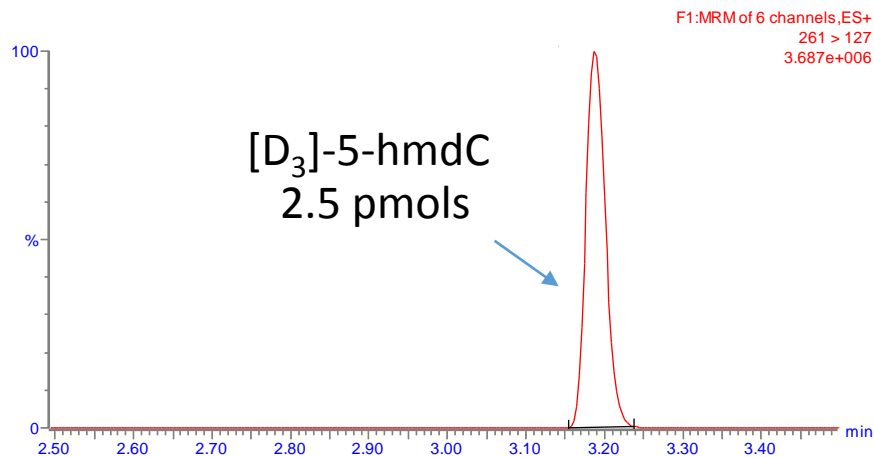

Supplement: S2 Fig — Sample chromatograms of extracted MRM traces for samples with medium (left side) and low (right side) concentrations of 5-hydroxymethyl-2’-deoxycytidine as well as 2.5 pmols of [D3]-5-hydroxymethyl-2’-deoxycytidine. (PDF) [file pone.0144859.s002.pdf]

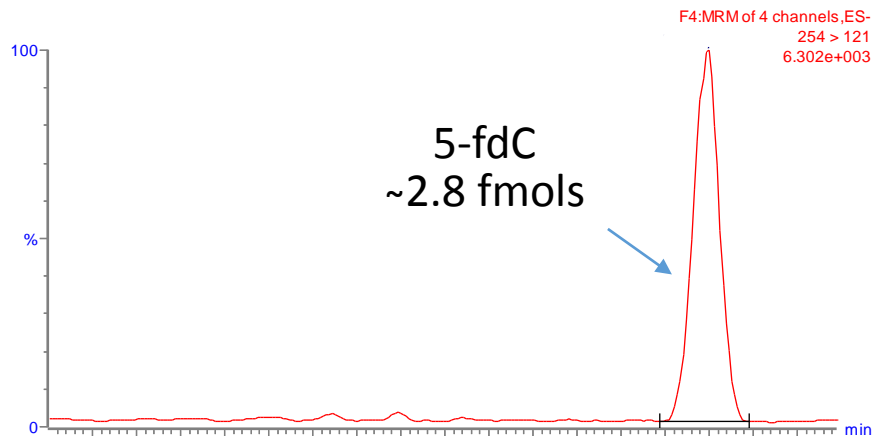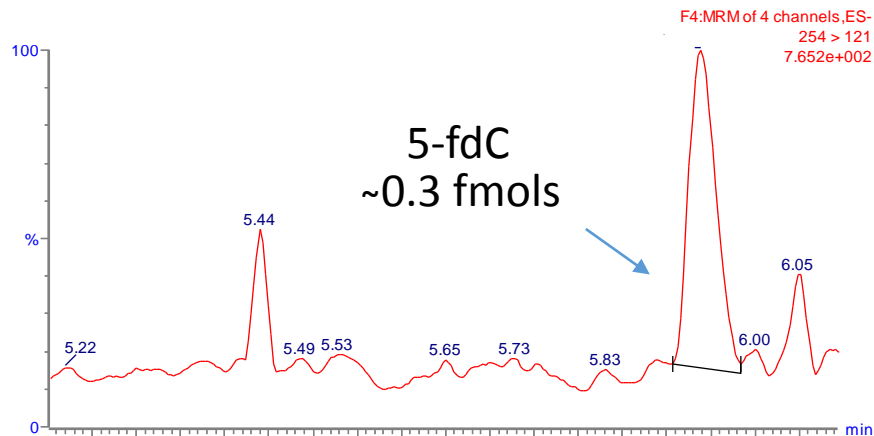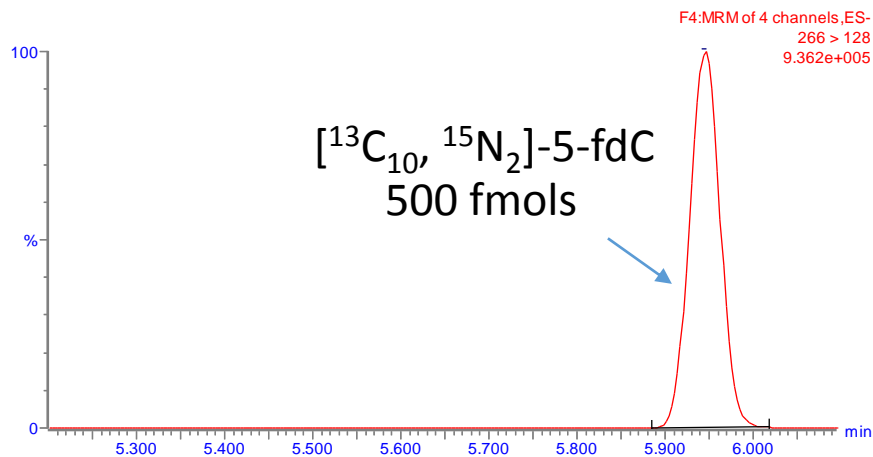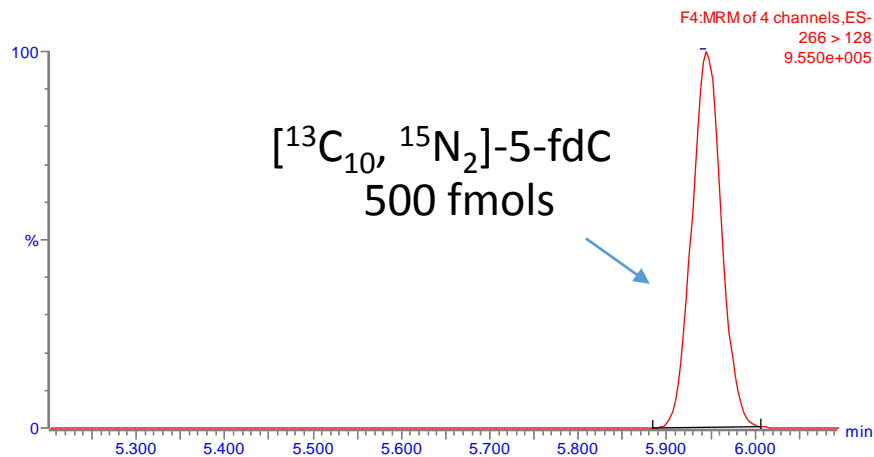

Supplement: S3 Fig — Sample chromatograms of extracted MRM traces for samples with medium (left side) and low (right side) concentrations of 5-formyl-2’-deoxycytidine as well as 500 fmols of [13C10, 15N2]-5-formyl-2’-deoxycytidine. (PDF) [file pone.0144859.s003.pdf]

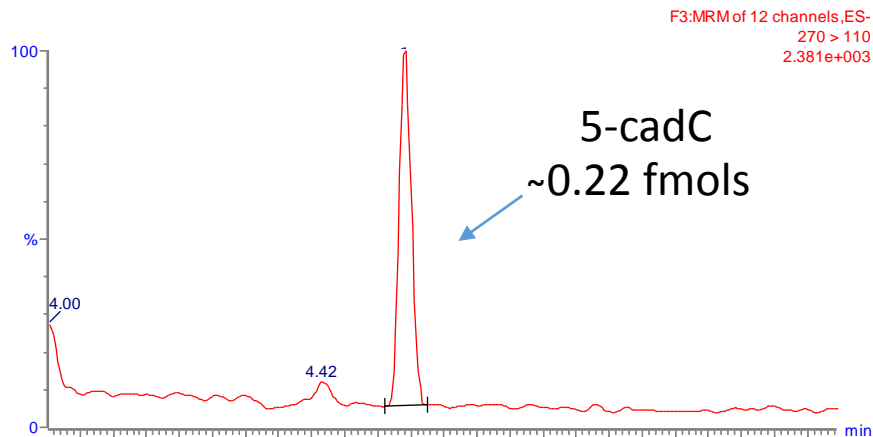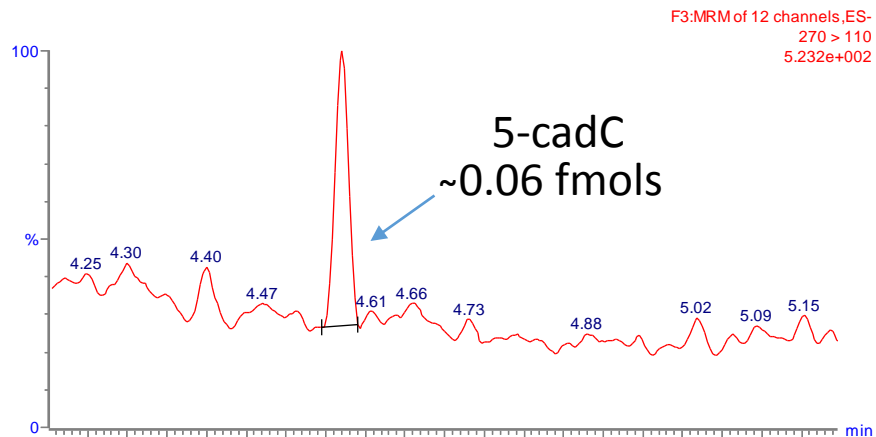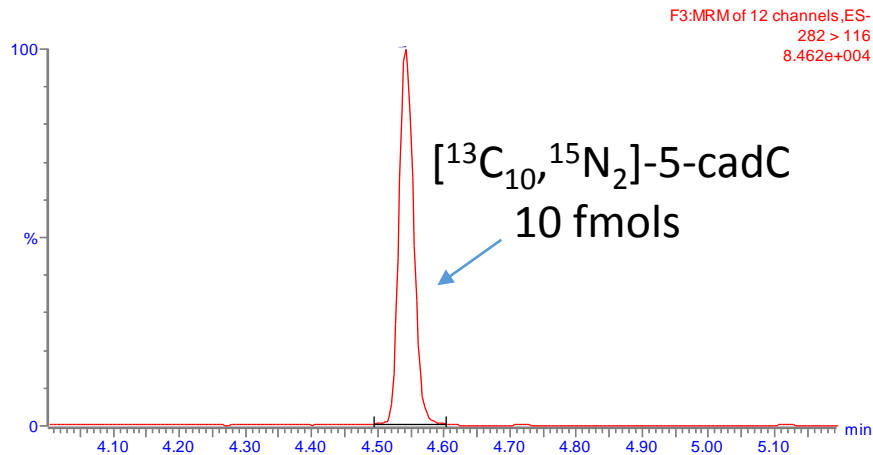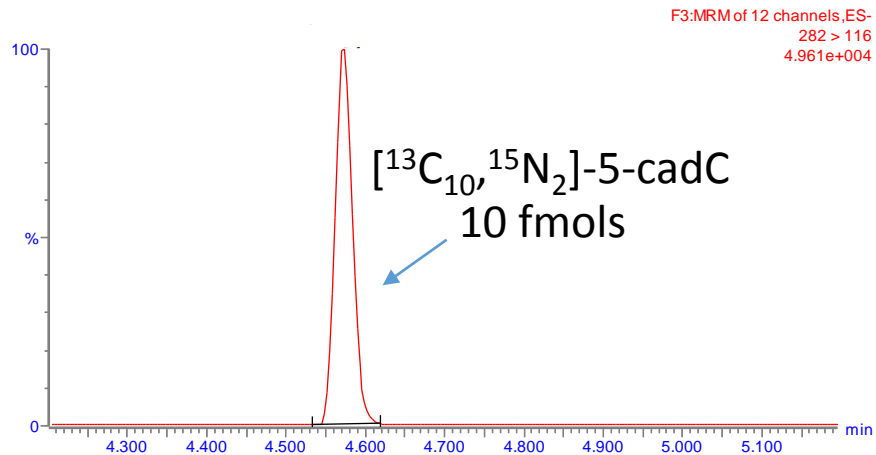

Supplement: S4 Fig — Sample chromatograms of extracted MRM traces for samples with medium (left side) and low (right side) concentrations of 5-carboxyl-2’-deoxycytidine as well as 10 fmols of [13C10, 15N2]-5-carboxyl-2’deoxycytidine. (PDF) [file pone.0144859.s004.pdf]

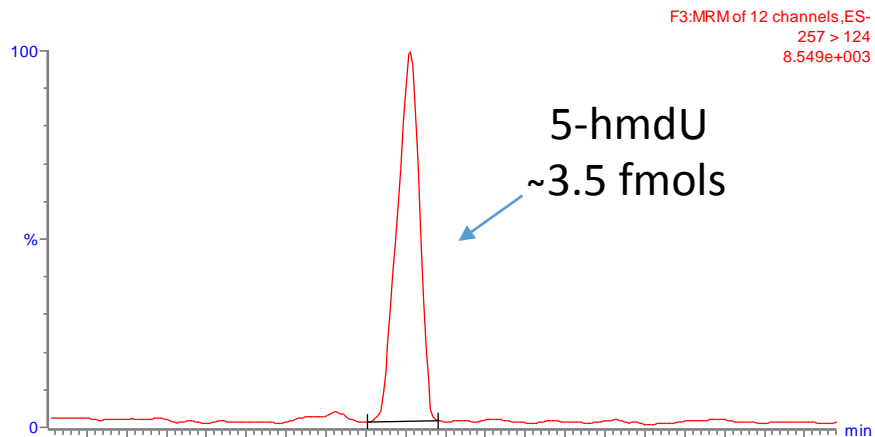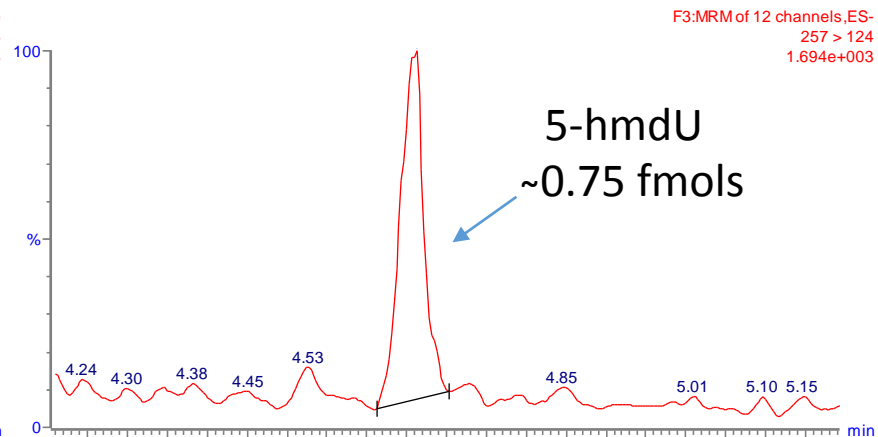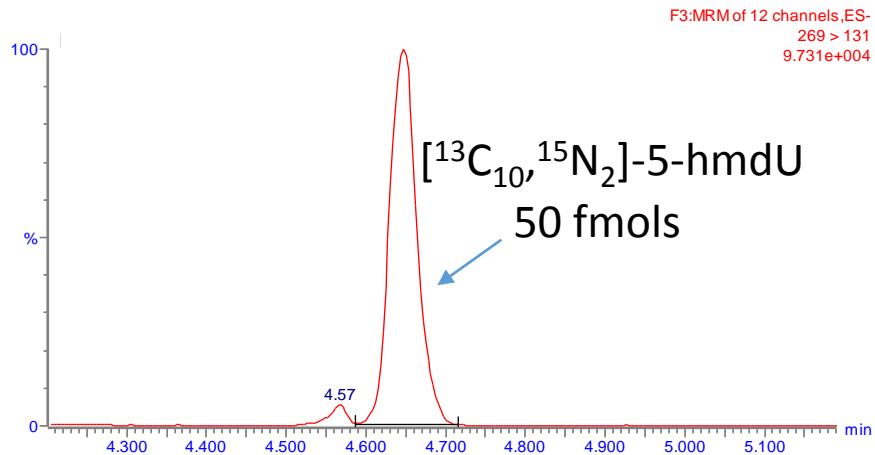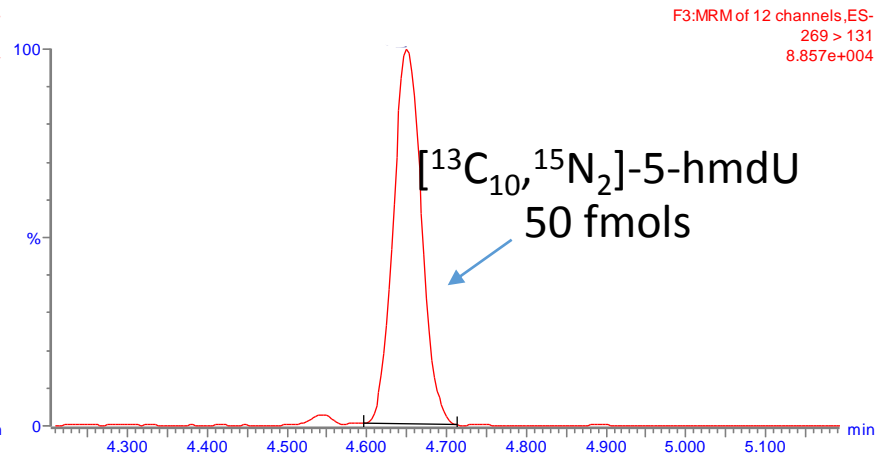

Supplement: S5 Fig — Sample chromatograms of extracted MRM traces for samples with medium (left side) and low (right side) concentrations of 5-carboxyl-2’-deoxycytidine as well as 50 fmols of [13C10, 15N2]-5 hydroxymethyl-2’-deoxyuridine. (PDF) [file pone.0144859.s005.pdf]

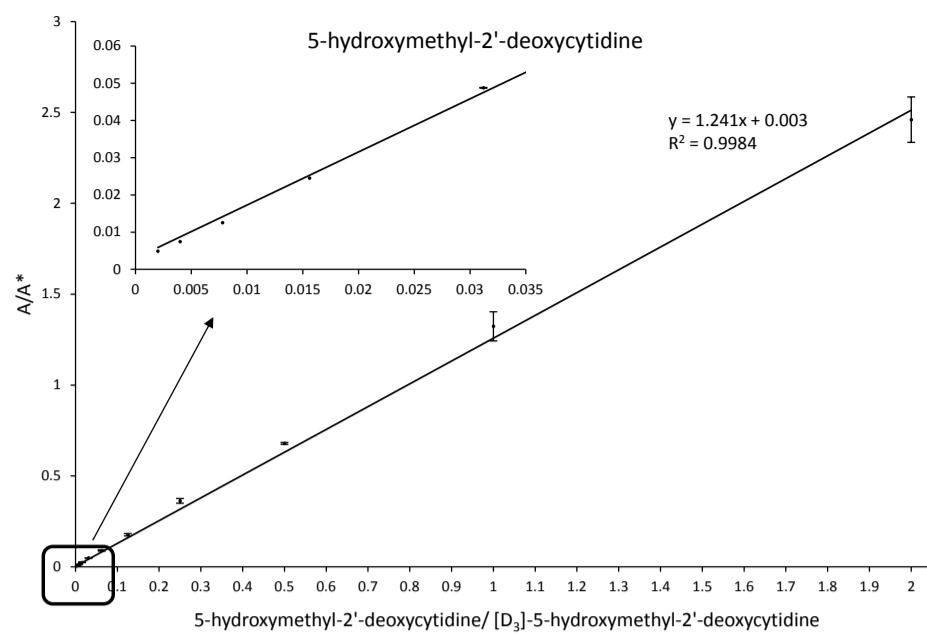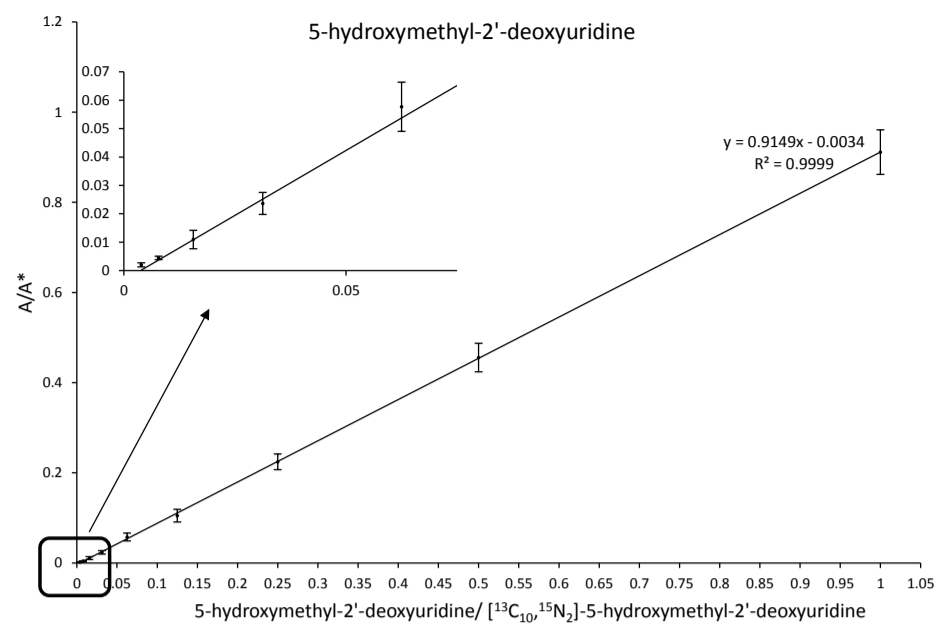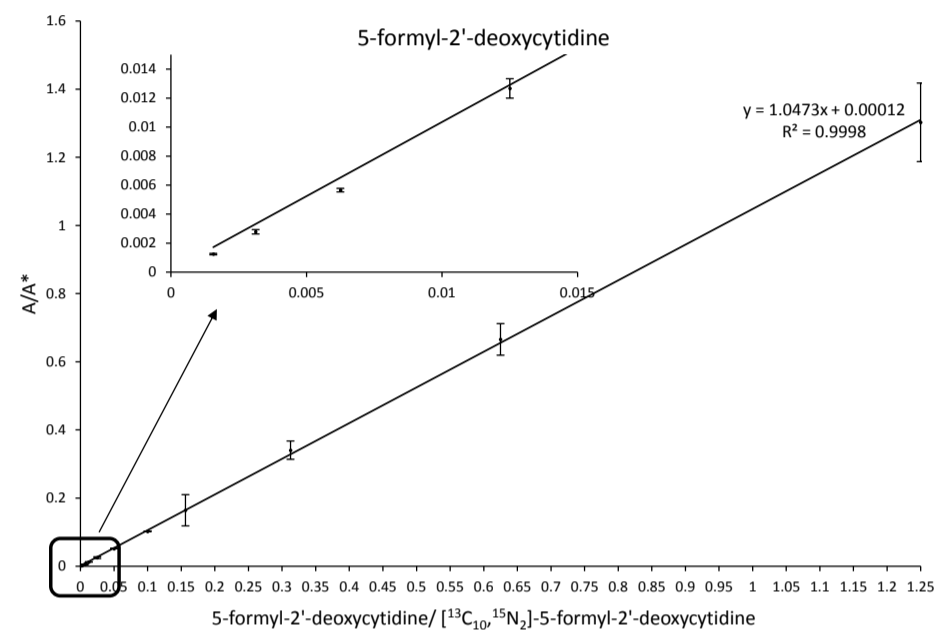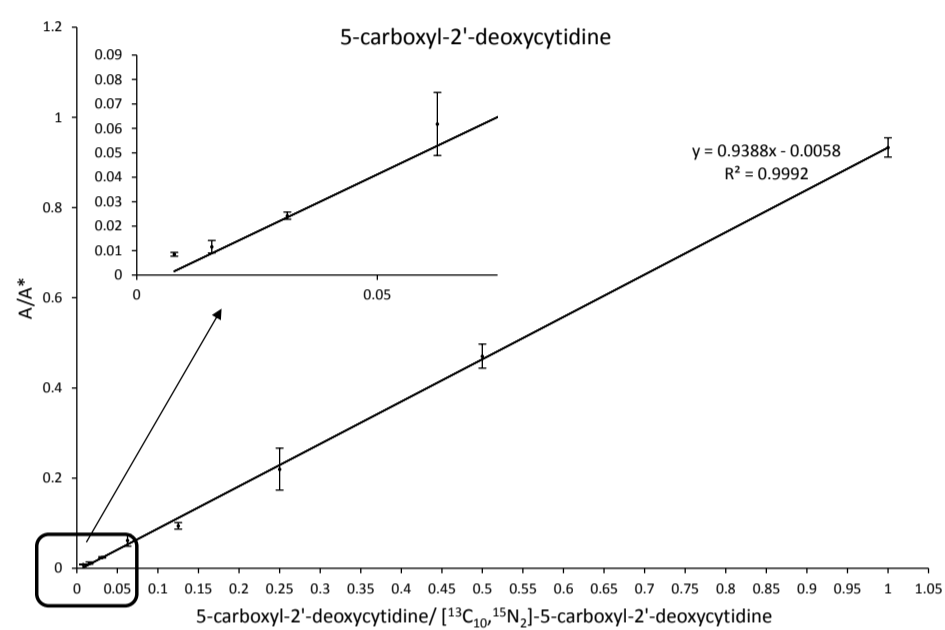

Supplement: S6 Fig — The curves were generated as correlation of detector response (defined as ratio of areas under peaks of non-labelled and stable isotope-labelled compound and molar ratio of compounds in the sample. Data presented as means for two injections; error bars represent standard deviations. Coefficients of determinations and p-values were calculated using Pearson’s analysis of correlation. (PDF) [file pone.0144859.s006.pdf]
