# Supplementary material for: Tissue-Specific Differences in DNA Modifications (5-Hydroxymethylcytosine, 5-Formylcytosine, 5-Carboxylcytosine and 5-Hydroxymethyluracil) and Their Interrelationships
Source: PLoS One. 2015 Dec 14;10(12):e0144859. doi: 10.1371/journal.pone.0144859 (PMC4682766; doi:10.1371/journal.pone.0144859)
Supplement: S1 Table — (PDF) [file pone.0144859.s007.pdf]

| | 5-mdC/$10^3$dN | 5-hmdC/$10^6$dN | 5-fdC/$10^6$dN | 5-cadC/$10^9$dN | 5-hmdU/$10^6$dN |
|---|---|---|---|---|---|
| pig liver 1 | 10.6828 | 0.1387 | 0.1415 | 8.3281 | 0.1824 |
| pig liver 2 | 10.1507 | 0.1913 | 0.0590 | 10.1569 | 0.1474 |
| pig liver 3 | 10.2292 | 0.1994 | 0.1148 | 14.7446 | 0.2776 |
| biological mean | 10.3542 | 0.1764 | 0.1051 | 11.0765 | 0.2025 |
| SD | 0.2872 | 0.0330 | 0.0421 | 3.3056 | 0.0674 |
| pig kidney 1 | 10.7153 | 0.2732 | 0.1019 | 7.4320 | 0.1038 |
| pig kidney 2 | 10.5613 | 0.3714 | 0.1435 | 3.1074 | 0.1143 |
| pig kidney 3 | 10.9557 | 0.2663 | 0.1334 | 5.5430 | 0.1585 |
| biological mean | 10.7441 | 0.3036 | 0.1263 | 5.3608 | 0.1255 |
| SD | 0.1988 | 0.0588 | 0.0217 | 2.1681 | 0.0290 |
| pig brain 1 | 10.9418 | 1.0762 | 0.5650 | 26.1457 | 0.1179 |
| pig brain 2 | 10.3273 | 1.5214 | 0.5194 | 13.4762 | 0.0977 |
| pig brain 3 | 10.8140 | 1.0007 | 0.5061 | 24.2853 | 0.1151 |
| biological mean | 10.6943 | 1.1994 | 0.5302 | 21.3024 | 0.1102 |
| SD | 0.3243 | 0.2814 | 0.0309 | 6.8412 | 0.0109 |
| pig thymus 1 | 12.7942 | 0.0442 | 0.1297 | 11.1940 | 0.1064 |
| pig thymus 2 | 12.5329 | 0.0548 | 0.1564 | 10.8747 | 0.1160 |
| pig thymus 3 | 12.2636 | 0.0549 | 0.1213 | 16.7455 | 0.0901 |
| biological mean | 12.5302 | 0.0513 | 0.1358 | 12.9381 | 0.1042 |
| SD | 0.2653 | 0.0061 | 0.0183 | 3.3012 | 0.0131 |
| pig lymph nodes 1 | 11.6343 | 0.0624 | 0.1198 | 3.5633 | 0.2564 |
| pig lymph nodes 2 | 11.9657 | 0.1236 | 0.3657 | 4.3521 | 0.9348 |
| pig lymph nodes 3 | 11.6006 | 0.1204 | 0.1648 | ND | 0.1784 |
| biological mean | 11.7336 | 0.1021 | 0.2168 | 3.9577 | 0.4565 |
| SD | 0.2017 | 0.0345 | 0.1309 | 0.5577 | 0.4160 |
| pig spleen 1 | 11.0769 | 0.1539 | 0.1465 | 13.6401 | 0.2864 |
| pig spleen 2 | 10.4086 | 0.1704 | 0.1470 | 5.5724 | 0.0868 |
| pig spleen 3 | 11.1787 | 0.1482 | 0.1266 | 10.6583 | 0.1562 |
| biological mean | 10.8881 | 0.1575 | 0.1401 | 9.9569 | 0.1765 |
| SD | 0.4184 | 0.0115 | 0.0116 | 4.0793 | 0.1013 |
| pig heart 1 | 11.1321 | 0.3285 | 0.2615 | 5.0737 | 0.3677 |
| pig heart 2 | 11.0876 | 0.3849 | 0.1426 | 6.2149 | 0.4135 |
| pig heart 3 | 10.9242 | 0.3796 | 0.1941 | 11.7955 | 0.3032 |
| biological mean | 11.0479 | 0.3643 | 0.1994 | 7.6947 | 0.3614 |
| SD | 0.1095 | 0.0312 | 0.0597 | 3.5969 | 0.0554 |
| pig intestine 1 | 11.1317 | 0.0972 | 0.3686 | 17.1524 | 0.6510 |
| pig intestine 2 | 11.6930 | 0.3014 | 0.5941 | 17.7913 | 0.5977 |
| pig intestine 3 | 11.3621 | 0.1547 | 0.3407 | 19.2655 | 0.6266 |
| biological mean | 11.3956 | 0.1844 | 0.4345 | 18.0697 | 0.6251 |
| SD | 0.2821 | 0.1053 | 0.1389 | 1.0837 | 0.0267 |
| pig muscle 1 | 9.4869 | ND | ND | ND | ND |
| pig muscle 2 | 9.6673 | ND | ND | ND | ND |
| pig muscle 3 | 9.9896 | 0.5387 | 0.3637 | 15.4300 | 0.1439 |
| biological mean | 9.7146 | 0.5387 | 0.3637 | 15.4300 | 0.1439 |
| SD | 0.2547 | NA | NA | NA | NA |
