# Supplementary material for: Tissue-Specific Differences in DNA Modifications (5-Hydroxymethylcytosine, 5-Formylcytosine, 5-Carboxylcytosine and 5-Hydroxymethyluracil) and Their Interrelationships
Source: PLoS One. 2015 Dec 14;10(12):e0144859. doi: 10.1371/journal.pone.0144859 (PMC4682766; doi:10.1371/journal.pone.0144859)
Supplement: S2 Table — (PDF) [file pone.0144859.s008.pdf]

	5-mdC/10^3dN	5-hmdC/10^6dN	5-fdC/10^6dN	5-cadC/10^9dN	5-hmdU/10^6dN
rat liver 1	8.2521	0.3517	0.5319	10.8022	ND
rat liver 2	8.3501	0.307370055	0.3939	10.6386	0.1095
biological mean	8.3011	0.3295	0.4629	10.7204	0.1095
SD	0.0693	0.0313	0.0976	0.1157	NA
rat kidney 1	7.7979	0.5062	0.2108	9.2897	0.2375
rat kidney 2	7.9689	0.5153	0.1974	3.6130	0.0755
rat kidney 3	7.8460	0.5494	0.2155	3.3701	0.1581
biological mean	7.8709	0.5236	0.2079	5.4243	0.1570
SD	0.0881	0.0227	0.0094	3.3498	0.0810
rat thymus 1	9.1926	0.0689	0.1648	3.8238	0.3098
rat thymus 2	9.0758	0.0606	0.1647	10.4073	0.3175
rat thymus 3	9.1001	0.0651	0.1720	ND	0.2039
biological mean	9.1229	0.0649	0.1672	7.1155	0.2770
SD	0.0616	0.0041	0.0042	4.6552	0.0635
rat heart 1	7.5124	0.4747	0.3525	7.5335	0.2997
rat heart 2	7.8495	0.4436	0.3168	6.8731	0.3093
rat heart 3	7.6944	0.4398	0.2845	ND	ND
biological mean	7.6854	0.4527	0.3179	7.2033	0.3045
SD	0.1687	0.0192	0.0340	0.4669	0.0068
rat intestine 1	7.7722	0.2320	0.3604	8.5387	0.3036
rat intestine 2	7.6411	0.1960	0.2787	14.4273	0.1188
rat intestine 3	7.9463	0.1810	0.3135	12.9575	0.1145
biological mean	7.7866	0.2030	0.3175	11.9745	0.1789
SD	0.1531	0.0262	0.0410	3.0649	0.1080
rat lung 1	7.8770	0.3592	0.2447	7.3627	0.1811
rat lung 2	7.7913	0.3610	0.2585	7.9899	0.1158
rat lung 3	8.0307	0.3212	0.2184	ND	0.1480
biological mean	7.8997	0.3471	0.2405	7.6763	0.1483
SD	0.1213	0.0225	0.0204	0.4435	0.0327
rat brain 1	8.6765	1.0017	0.7947	34.7908	0.1090
SD (techn. replicates)	0.2918	0.0170	0.1867	2.1416	0.0630
